# A pyrazolopyran derivative preferentially inhibits the activity of human cytosolic serine hydroxymethyltransferase and induces cell death in lung cancer cells

**DOI:** 10.18632/oncotarget.6726

**Published:** 2015-12-22

**Authors:** Marina Marani, Alessio Paone, Alessio Fiascarelli, Alberto Macone, Maurizio Gargano, Serena Rinaldo, Giorgio Giardina, Valentino Pontecorvi, David Koes, Lee McDermott, Tianyi Yang, Alessandro Paiardini, Roberto Contestabile, Francesca Cutruzzolà

**Affiliations:** ^1^ Department of Biochemical Sciences “A. Rossi Fanelli”, Sapienza University of Rome, Rome 00185, Italy; ^2^ Department of Computational and Systems Biology, University of Pittsburgh, Pittsburgh, PA 15213, USA; ^3^ Department of Pharmaceutical Sciences and Drug Discovery Institute, University of Pittsburgh, Pittsburgh, PA 15261, USA; ^4^ Department of Chemistry and Biochemistry Cristol 63, University of Colorado, Boulder, CO 80302, USA; ^5^ Department of Biology and Biotechnology “Charles Darwin”, Sapienza University of Rome, Rome 00185, Italy

**Keywords:** lung cancer, serine hydroxymethyltransferase, pyrazolopyrans, inhibition, apoptosis

## Abstract

Serine hydroxymethyltransferase (SHMT) is a central enzyme in the metabolic reprogramming of cancer cells, providing activated one-carbon units in the serine-glycine one-carbon metabolism. Previous studies demonstrated that the cytoplasmic isoform of SHMT (SHMT1) plays a relevant role in lung cancer. SHMT1 is overexpressed in lung cancer patients and NSCLC cell lines. Moreover, SHMT1 is required to maintain DNA integrity. Depletion in lung cancer cell lines causes cell cycle arrest and uracil accumulation and ultimately leads to apoptosis. We found that a pyrazolopyran compound, namely 2.12, preferentially inhibits SHMT1 compared to the mitochondrial counterpart SHMT2. Computational and crystallographic approaches suggest binding at the active site of SHMT1 and a competitive inhibition mechanism. A radio isotopic activity assay shows that inhibition of SHMT by 2.12 also occurs in living cells. Moreover, administration of 2.12 in A549 and H1299 lung cancer cell lines causes apoptosis at LD50 34 μM and rescue experiments underlined selectivity towards SHMT1. These data not only further highlight the relevance of the cytoplasmic isoform SHMT1 in lung cancer but, more importantly, demonstrate that, at least *in vitro*, it is possible to find selective inhibitors against one specific isoform of SHMT, a key target in metabolic reprogramming of many cancer types.

## INTRODUCTION

A major feature distinguishing cancer cells from non-malignant cells is their ability to grow and divide uncontrollably. To deal with the increasing needs caused by active proliferation, cells shift their metabolism toward aerobic glycolysis to enhance production of biosynthetic intermediates. Indeed, in the last few years, reprogramming of cellular metabolism has been recognized as a hallmark of cancer [1]. In a subset of human tumors, including melanoma, breast and non-small-cell lung cancer (NSCLC), a significant amount of the glycolytic carbon is redirected into the synthesis of serine. Serine anabolism then fuels the *de novo* biosynthesis of purines and pyrimidines and the production of antioxidant molecules [2–4]. Thus, serine/glycine one-carbon (SGOC) metabolism and, in particular, serine hydroxymethyltransferase (SHMT), the enzyme providing activated one-carbon units by converting serine and tetrahydrofolate (H_4_PteGlu) to glycine and 5, 10-CH_2_-H_4_PteGlu (ME-THF), represent focal points of the metabolic reprogramming of cancer cells.

In humans, two SHMT genes are found: *SHMT1*, which encodes the cytoplasmic isozyme (SHMT1) and *SHMT2*, encoding the mitochondrial one (SHMT2) [5]. These two isoforms display a 66% amino acid sequence identity. *SHMT2* also encodes a second transcript SHMT2α that lacks the mitochondrial import signal, and is thus localized in the cytoplasm [6]. SHMT2 seems preferentially involved in the synthesis of glycine and mitochondrial dTMP [7, 8], while SHMT1 and, to a lower extent (25%), SHMT2α participate to the synthesis of dTMP, undergoing nuclear import during S-phase and supplying ME-THF during the thymidylate cycle, along with thymidylate synthase (TS) and dihydrofolate reductase (DHFR) [9]. SHMT2 has been recently shown to be upregulated under hypoxic conditions [10], producing glycine and ME-THF and thereby increasing the synthesis of NADPH, which is necessary to counteract the increase in oxidative stress experienced under low oxygen tension.

*SHMT1* polymorphisms have been associated with increased lung cancer risk [11]. We recently demonstrated that SHMT1 plays a relevant role in lung cancer, as it is overexpressed in tissue samples from lung cancer patients and NSCLC cell lines. Moreover, knockdown of SHMT1 in lung cancer cells triggers cell cycle arrest and, during DNA replication, uracil accumulation causing apoptosis in a p53-dependent manner. Therefore, nuclear localization of SHMT1 is required to maintain DNA integrity [12].

Lung cancer remains the most common cancer in the world, both in term of new cases and deaths because of the high case fatality [13]. The role played by SHMT at the crossroads of different key metabolic pathways (serine/glycine and nucleotide/folate metabolism) makes it a potential target of novel chemotherapeutic drugs [14–16]. Despite its relevance, only a few studies that focus on drug design strategies and discovery of compounds that can inhibit SHMT have been carried out to date. The search for selective serine analogues and amino acid derivatives as SHMT inhibitors has not been successful [17].

Antimetabolites, the drugs quenching the effects of metabolites on cellular processes, are a landmark in anticancer therapy. The only antifolate compounds with anticancer activity found to inhibit SHMT, apparently irreversibly, were the quite toxic sulphonyl fluoride triazine derivatives [18]. Leucovorin (5-CHO-H_4_PteGlu) has been indicated as another inhibitor of both SHMT isoforms, with preference for SHMT1 over SHMT2. Unfortunately, it cannot be used clinically as an SHMT inhibitor, as it is converted to other folic acid derivatives (e.g., H_4_PteGlu) and thus has vitamin activity, equivalent to that of folic acid [19]. We have recently identified two other antifolates, pemetrexed [20] and lometrexol [21], which act *in vitro* as micromolar inhibitors of SHMT. However, these are both multitarget antifolates, approved by the US Food and Drug Administration (FDA) for the treatment of mesothelioma (in combination with cisplatin) and NSCLC.

**Figure 1:**
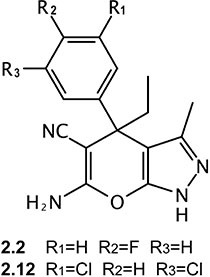
Chemical structure of compounds 2.2 and 2.12

A novel set of 338 molecules sharing a pyrazolopyran scaffold were recently reported in a patent application by BASF AG (WO 2013182472 A1) as plant SHMT inhibitors with IC_50_ values in the low micromolar/nanomolar range. Given the key role played by plant SHMT in the photorespiration cycle, controlling the formyl-transfer between glycine and serine, these compounds were suggested primarily as weed killers, but have been recommended also as pharmaceutically active ingredients for treating or preventing parasitic and/or bacterial infections. More recently, it was shown that some of those pyrazolopyrans are also active against the *Plasmodium falciparum* SHMT protein [23]. These two facts prompted us to test a low and a high activity compound from the WO 2013182372 A1 patent application against human SHMTs with the objective of performing preliminary assessment of the activity and potential of this pyrazolopyran class as human SHMT inhibitors. We randomly selected compounds 2.2 and 2.12 (Figure 1) for further experimentation. Here we demonstrate that, unlike compound 2.2 that shows a low affinity for both human SHMTs, compound 2.12 ((4R)-6-amino-4-ethyl-4-(3, 5-chlorophenyl)-1H-pyrano[2,3-c]pyrazole-5-carbonitrile) is able to selectively inhibit the human cytoplasmic isoform of SHMT versus the mitochondrial one. In addition, treatment of lung cancer cells with compound 2.12 induces cell death through the activation of apoptosis. Our data open the possibility of designing a new generation of effective chemotherapeutic agents acting as selective SHMT inhibitors based on this scaffold.

## RESULTS

### Inhibition of SHMT cytosolic and mitochondrial isoforms by compounds 2.2 and 2.12

**Figure 2:**
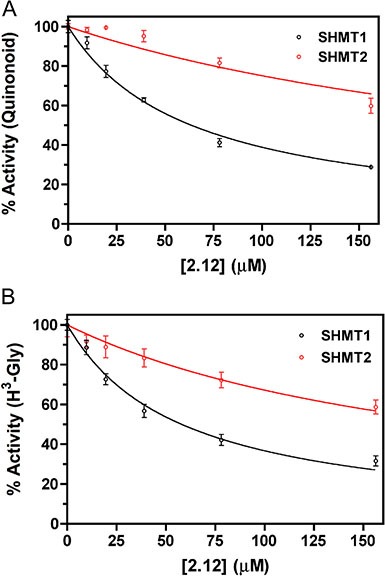
Activity inhibition on purified SHMTs by compound 2.12 The activity of cytosolic (black symbols) and mitochondrial (red symbols) SHMT isoforms was measured in the presence of increasing concentrations of compound 2.12. Values are the average ± standard deviation of three independent measurements. Continuous lines through the experimental points are those obtained from the least square fitting of data to equation 1. Two different competitive binding assays were carried out. (**A**) One assay was based on the spectrophotometric measurement of the quinonoid intermediate that develops when both glycine and H_4_PteGlu are bound to the enzyme. Estimated inhibition constants were 57.9 ± 5.5 μM for SHMT1 and 227.2 ± 38.0 μM for SHMT2. (**B**) The results shown are based on a radioisotope assay that exploits the capability of SHMT to catalyse the exchange of glycine 2-pro-S proton with the solvent. With this assay, the calculated inhibition constants were 59.6 ± 4.6 μM for SHMT1 and 252.8 ± 30.7 μM for SHMT2.

Initially, compounds 2.2 and 2.12 (Figure 1) were tested on purified recombinant SHMT1 and SHMT2 isoforms using a spectrophotometric assay, in which the inhibitors and H_4_PteGlu compete for binding to the same site of the enzyme [20, 21]. Both isozymes (5.5 μM) were incubated with a saturating concentration of glycine (3.0 mM), in the presence of 20 μM H_4_PteGlu and a varying concentration of either compound 2.12 or 2.2. The activity of the enzyme was calculated from the spectrophotometric measurement of the quinonoid intermediate that develops when both glycine and H_4_PteGlu bind to SHMT [20]. The estimated inhibition constants (IC_50_) were: for SHMT1, 154.5 ± 14.4 μM with compound 2.2 (Supplemental Figure 1) and 57.9 ± 5.5 μM with compound 2.12 (Figure 2A); for SHMT2, 262.8 ± 48.0 μM with compound 2.2 (Supplementary Figure 1) and 227.2 ± 38.0 μM with compound 2.12 (Figure 2A). This encouraging result with compound 2.12 was confirmed in a second inhibition assay using a radioisotopic method, with known specificity for SHMT [12], and which measures the rate of exchange of tritium from H^3^-glycine to water. In this assay, the enzyme concentration was 43 nM, H_4_PteGlu was 20 μM, glycine was 23 μM and compound 2.12 concentration varied between 9.75 μM and 156 μM. Calculated inhibition constants in the latter assay were 59.6 ± 4.6 μM for SHMT1 and 252.8 ± 30.7 μM for SHMT2 for 2.12 (Figure 2B). Considering the ability of 2.12 to selectively target human SHMT1 and the high inhibition constant of 2.2, we decided to focus our attention on 2.12 in subsequent experiments.

### Predicted binding mode of 2.12 to human SHMT1

**Figure 3:**
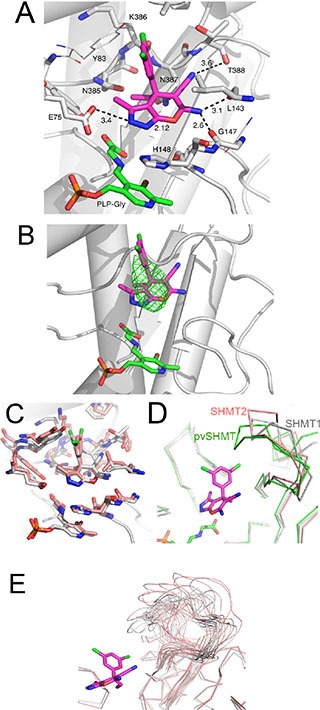
Template-based docking of 2.12 into the active site of SHMTs (**A**) Docked 2.12 to human SHMT1, 2.12 and PLP-Glycine complex are depicted as purple and green sticks, respectively. Protein-ligand polar interactions are shown as black dashes, distances are given in Å. Residue numbering refers to SHMT1. (**B**) Docked 2.12 superposed with the low-resolution (4.2 Å) difference electron density map (contour level at 3.0 sigma) of SHMT1 co-crystallized with 2.12. The difference electron density map is shown as green mesh. (**C**) Comparison of 2.12 docked to SHMT1 (white) and pvSHMT (pink). Atom coloring is the following: N blue, O red, P orange. The backbone of SHMT1 is also shown, as reference. (**D**) Comparison of the loop region (residues 386-399) of human SHMT1 (white), *Plasmodium vivax* SHMT (green) and human SHMT2 (mitochondrial, pink). Only Cα traces are shown. 2.12 is also shown, as pink sticks. (E) Loop conformations sampled during a 100 ns molecular dynamics simulation of apo human SHMT1 (white) and SHMT2 (pink).

Based on the recently solved crystal structure of *Plasmodium vivax* SHMT in complex with the pyrazolopyran compound 5-{3-[(4S)-6-amino-5-cyano-3-methyl-4-(propan-2-yl)-2, 4-dihydro pyrano[2, 3-c]pyrazol-4-yl]-5-cyanophenyl}thiophene-2-carboxylate ([23] PDB: 4TMR), a template-based docking of 2.12 was carried out into the active site of human SHMT1 (Figure 3A). Compound 2.12, like the pyrazolopyran in the 4TMR crystal structure, is predicted to occupy the H_4_PteGlu binding site of human SHMT1. The binding location of 2.12 was confirmed via low-resolution crystallographic data (4.2 Å resolution) of human SHMT1 in complex with 2.12, which showed a positive electron density, up to 5.0 sigma, in agreement with the docking results (Figure 3B). According to the docking results, 2.12 interacts with human SHMT1 showing a net preference for the (*S*)-absolute configuration, as also observed in *Plasmodium vivax* SHMT [23] (Figure 3C).

In our model, a polar environment encloses the pyrazolopyran moiety of 2.12. The backbone of Thr388 forms a hydrogen bond with the cyano group and a bidentate interaction is formed between the amino group of 2.12 and the backbone carbonyls of Leu143 and Gly147. The N2 of the pyrazole moiety undergoes hydrogen bonding to Glu75. A small pocket formed by residues Asn385-Asn387 is filled by the ethyl group at the chiral center of 2.12. The 3, 5-substituted phenyl group consists of two hydrophobic Cl atoms, one pointing to a small lateral pocket and making contact with Tyr83 and Lys386, and the other one further extending along the active site channel and engaging in nonpolar interactions along the channel.

### Involvement of a flexible loop in 2.12 binding

The IC_50_ of 2.12 for *Plasmodium falciparum* SHMT is 280 nM ([23]; compound number 44 in table S1 of Supporting Information). In order to derive a structure-based rationale for the observed difference of affinity displayed by 2.12 for human and *Plasmodium* SHMT, we compared the binding mode of 2.12 to the active sites of both enzymes, using as reference the crystal structure of *Plasmodium vivax* SHMT in complex with a pyrazolopyran compound ([23] PDB: 4TMR; 83% identity with *Plasmodium falciparum* SHMT) (Figure 3C). As SHMT is an enzyme of pivotal importance for nucleotide synthesis, it is not surprising that its overall structure, and in particular the residues composing the active site, are well conserved through evolution. Residues of *Plasmodium* SHMT binding to the pyrazolopyran scaffold [23] are invariant in human SHMT1 and SHMT2. The only exception is Thr183 of *Plasmodium* SHMT, which is conservatively replaced by Ser203 in human SHMT1 (Ser205 in SHMT2). The difference of affinity displayed by 2.12 for human and *Plasmodium falciparum* SHMTs may in part be due to this Ser/Thr substitution. Another key structural difference between *Plasmodium vivax* SHMT and human SHMT1 lies in the loop region formed by the residues Asn387-Ser399 in human SHMT1 and the equivalent residues Asn356-Ser368 in *Plasmodium vivax*. This region, which forms part of the THF binding cleft, appears to be flexible and has a different conformation in these structures (Figure 3D). In the docked model, the *Plasmodium vivax* SHMT Asn356-Ser368 loop enclose the hydrophobic Cl atoms of 2.12 in an apolar environment (which also includes Tyr63) more effectively than the human SHMT1 and SHMT2 loop. The different residue composition and conformation that this loop assumes may explain, from a structural point of view, the difference in affinity observed for 2.12. The dynamics of this loop region may also contribute: in molecular dynamics simulations, this loop displays slightly greater flexibility in SHMT2, as measured by the root mean squared deviation in atomic coordinates from the initial structure. Increased flexibility in the unbound structure implies an increased entropic penalty upon stabilization by ligand binding. Perhaps more importantly, as shown in Figure 3E, the two isoforms sample a different conformational space of this loop, with SHMT1 displaying a greater tendency towards enclosing the binding pocket. This indicates that in SHMT1 this loop may be predisposed toward conformations that interact with the dichlorobenzene ring that distinguishes 2.12 from 2.2.

### Inhibition of SHMT activity in living cells

**Figure 4:**
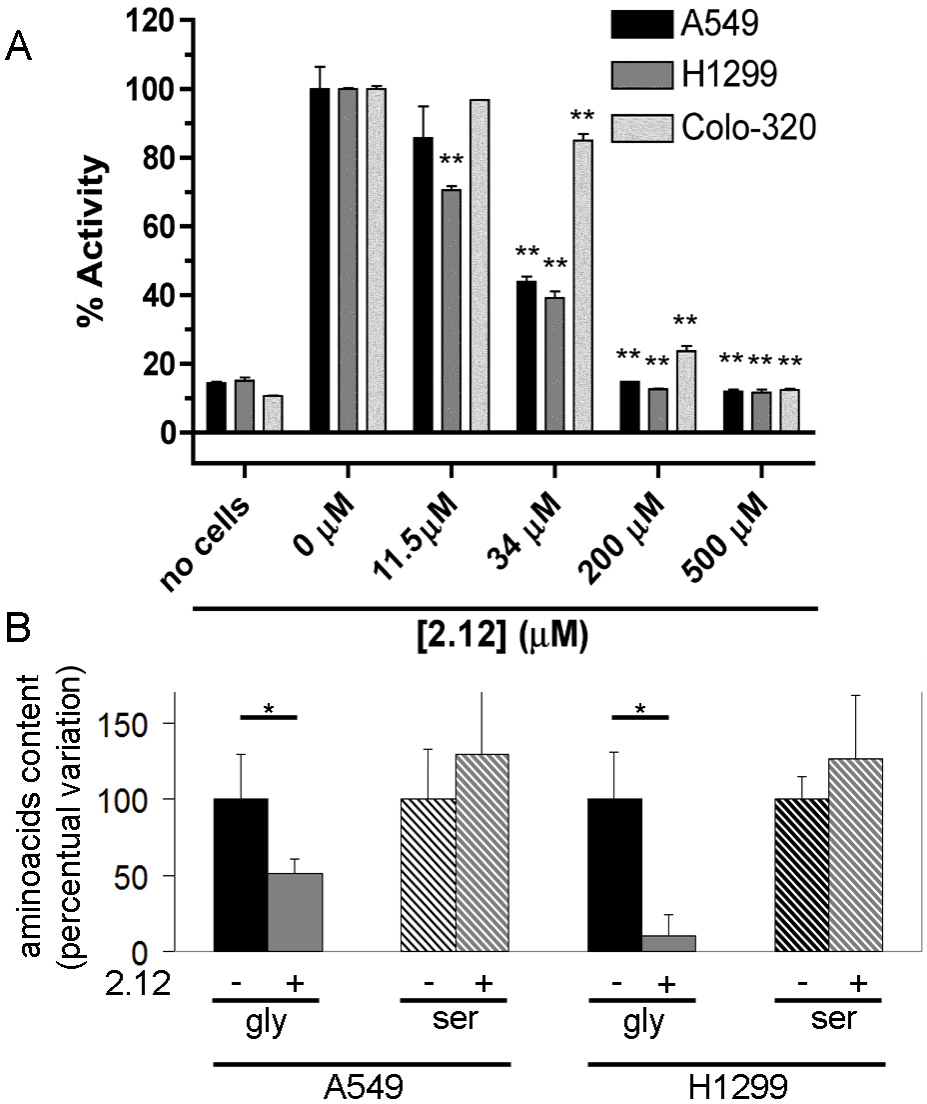
Inhibition of SHMTs activity in living cells (**A**) SHMTs activity was measured in A549 and H1299 lung cancer cell lines and COLO320 colon cancer cell line after treatment with 2.12 at different concentrations. The inhibitory effect of 2.12 occurs in a dose dependent manner. The histogram represents mean ± standard deviation of two independent replicates. (** = *P* ≤ 0.001). (**B**) Gas-chromatography/mass-spectrometry semi quantitative evaluation of glycine and serine content in A549 and H1299 cells treated or not with 2.12 (25 μM for 24 h). The mean ± standard deviation of three independent experiments is shown in the histogram (* = *P* ≤ 0.05).

Several recent papers describe SHMT2 upregulation in several cancer cell types, including colon and breast [24, 25], underlying the relevance of this isoform in the metabolism of specific cancer cells, where it is upregulated possibly to counteract redox stress. On the other hand, we recently demonstrated the crucial role of SHMT1 for the survival of lung cancer cells; albeit being overexpressed also in these cells [12], SHMT2 appears to play a minor role. These data suggest that the two SHMT isoforms may have distinct functions in different types of tumours. To test the ability of compound 2.12 to inhibit SHMT in cancer cells, we measured the SHMT activity in living cells by a modified version of the above-described radioisotopic assay [26]. Together with lung cancer cell lines A549 and H1299, which over-express both SHMT1 and SHMT2 [12], we also used COLO320 cells that over-express SHMT2 but not SHMT1 (Supplemental Figure 2). The cultured cells were pre-treated with 2.12 or DMSO (the solvent in which 2.12 was dissolved) for 1 hour. Figure 4A shows that 2.12 inhibits the total SHMT activity in cancer cell lines in a dose dependent manner and that SHMT activity in lung cancer cells is much more sensitive to compound 2.12 (IC_50_ between 11.5 and 34 μM) than in colon cancer cells (IC_50_ between 34 and 200 μM). These data are in agreement with the higher affinity of 2.12 for SHMT1 observed in tests with purified proteins.

To validate the ability of 2.12 to inhibit SHMT1 in lung cancer cells, we used gas chromatography/mass spectrometry to evaluate the glycine and serine content in A549 and H1299 cells treated or not with 2.12. Data in Figure 4B show a strong decrease in glycine content in both cells lines after 2.12 treatment. The levels of serine seem to be slightly increased but this variation is not statistically significant. Overall, data suggest a direct effect of 2.12 compound on SHMT(s) in living cells.

### Treatment with 2.12 induces cell death in cancer cells

We previously demonstrated that RNA interference (RNAi) against SHMT1 induces apoptosis in lung cancer cell lines [12], via the misincorporation and accumulation of uracil in DNA. To test the ability of the SHMT inhibitor 2.12 to induce cell death, we treated the A549, H1299 and COLO320 cells with different doses of 2.12 for 24 h, 48 h and 72 h and performed trypan blue exclusion and XTT assays.

**Figure 5:**
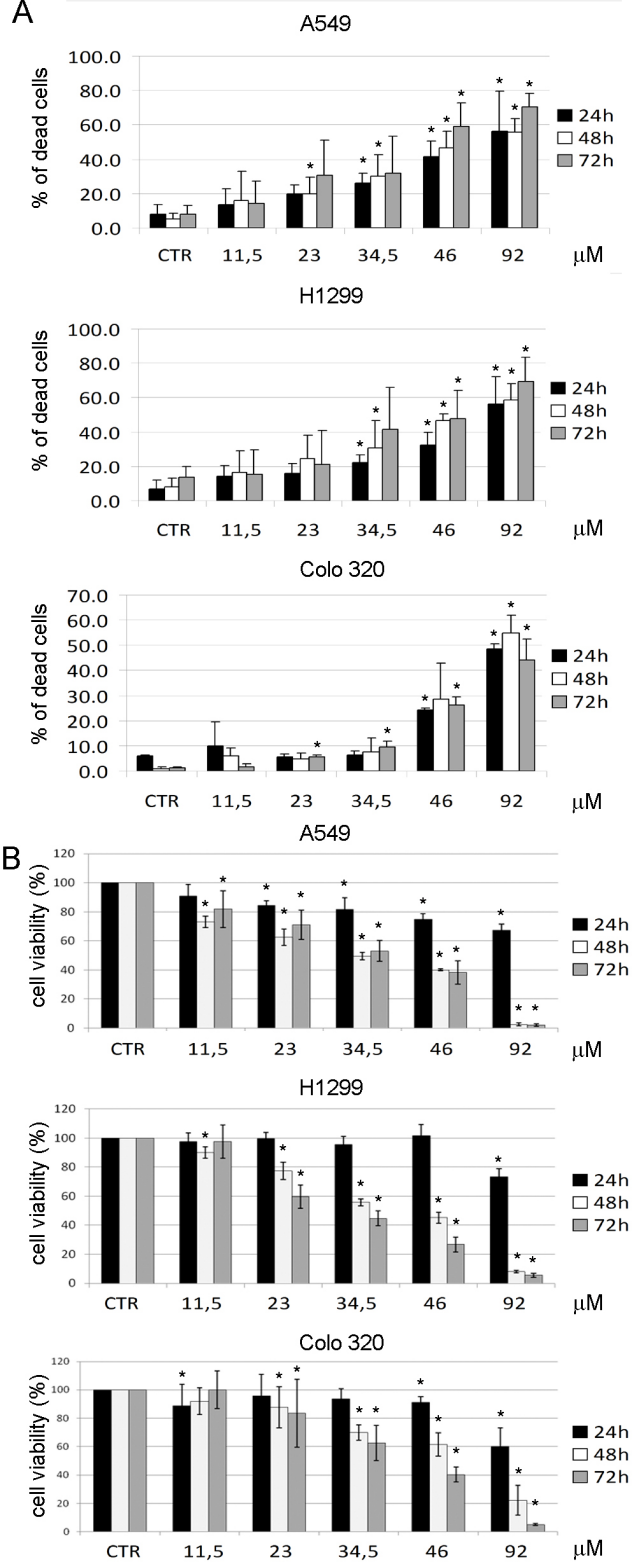
Cell death induction after treatment with 2.12 A549, H1299 and COLO320 cancer cell lines were treated for 24 h, 48 h and 72 h with increasing concentrations of 2.12 and cell death was evaluated by trypan blue exclusion assay (**A**) and XTT assay (**B**). All data represent mean ± standard deviation of at least 3 independent experiments. (* = *P* ≤ 0.05)

Figure 5 shows that 2.12 induces cell death in all the cell lines tested, as evident in both the trypan blue exclusion assay, which evaluate dead cells by looking at cell membrane integrity (Figure 5A) and the XTT, which highlights cells with active mitochondria (Figure 5B). Both assays, although with slightly different numbers due to the different experimental approaches, indicate that the Lethal Dose_50_ (LD_50_) for lung cancer cells is around 34 μM, while the LD_50_ for COLO320 cells is between 46 and 92 μM. These data demonstrate that a lower concentration of 2.12 is needed to kill A549 and H1299 cells compared to the COLO320 cell line.

### 2.12 induces cell death through an apoptotic process

**Figure 6:**
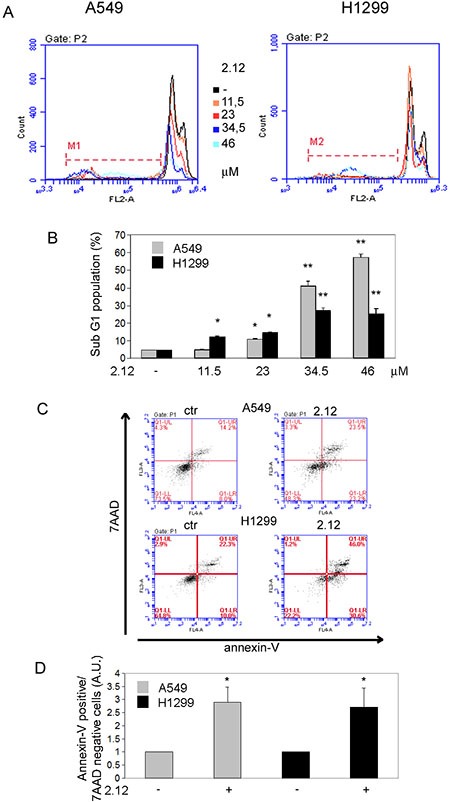
Activation of apoptotic process causes 2.12-mediated cell death Apoptotic induction analysis by (**A** and **B**) PI staining after 2.12 treatment at different concentrations for 48 h and (**C** and **D**) AnnexinV/7-ADD staining after 2.12 treatment at 34 μM for 48 h in A549 and H1299 cell lines. Data in B and D represent mean ± standard deviation of at least 3 independent replicates. * = *P* ≤ 0.05, * = *P* ≤ 0.01.

To clarify if the 2.12-mediated induction of lung cancer cell death is due to the activation of an apoptotic process, we used PI and Annexin-V staining. Figure 6A and 6B shows that 2.12 induces the accumulation of a subG1 population of A549 and H1299 lung cancer cells after 48 hours of treatment, clearly indicating an apoptotic phenomenon, with a strong induction (around 40% for A549 and 25% for H1299) at the concentration of 34 μM. Figure 6C and 6D shows the same cell lines after treatment with 2.12 (34 μM for 48 h) together with the control untreated samples, both stained with annexin-V. The accumulation of cells in the population Annexin-V positive/7-AAD negative after treatment with 2.12 confirms that the apoptotic mechanism is involved.

### Rescue of 2.12-treated lung cancer cells by metabolites providing one-carbon units

**Figure 7:**
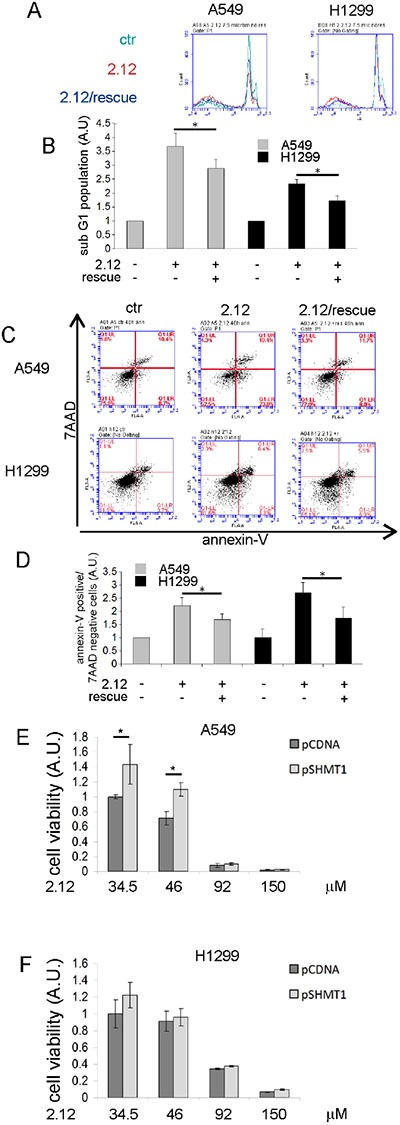
Effect of selected metabolites on cells 2.12-treated and selectivity of 2.12 against SHMT1 Apoptotic rate evaluation of A549 and H1299 treated or not with 2.12 and grown in minimal essential medium supplemented or not with serine, glycine and 5-formyl-THF. (**A**) and (**B**) PI staining, (**C**) and (**D**) AnnexinV/7-ADD staining. (**E**) and (**F**) XTT assay on A549 and H1299 respectively transfected with the indicated plasmid and treated or not with 2.12 34 μM for 48 hours. Data in B, D, E and F represent mean ± standard deviation of at least 3 independent experiments. * = *P* ≤ 0.05.

It is well known that adding selected metabolites can rewire the cellular metabolism, thus bypassing deficiencies in a given enzyme, as previously shown for SHMT2 [25]. We employed a similar approach to evaluate whether the apoptotic effect induced by compound 2.12 was due to the inhibition of the SHMT SER/GLY activity. The 2.12-treated (34 μM) or untreated A549 and H1299 lung cancer cells were grown in a minimal medium supplemented or not with serine, glycine and 5-formyl-THF (used in these experiments for its higher stability compared to THF) [21]. The apoptotic rate was evaluated using PI and annexin-V/7AAD staining. The data in Figure 7A and 7B (PI staining) and 7C and 7D (Annexin-V/7AAD staining) demonstrate that the 2.12-induced apoptotic effect is partially rescued (∼ 28%) if the cells are grown in the supplemented medium, suggesting that SHMT SER/GLY activity is likely involved in the observed apoptotic effect.

To validate the selectivity of 2.12 for SHMT1 we used a parallel strategy, involving the over-expression of this protein in the selected cell lines starting from a plasmid-borne gene; SHMT1 overexpression was confirmed by western blot (Supplemental Figure 3). The effect of compound 2.12 (34 μM) on cells transfected with the plasmid encoding SHMT1 was evaluated by studying cell viability (XTT assay). Figure 7E shows that SHMT1 overexpression is able to partially rescue (∼30%) the death effect induced in A549 cells by treatment with 2.12 (34.5 and 46 μM) indicating that SHMT1 is a direct target of 2.12. Unfortunately, when using H1299 cells in the same experiment, we did not reach statistical significance despite several repeats, although we observed a similar trend (Figure 7F). We have previously shown that the expression of SHMT1 and SHMT2 are interconnected and there are differences in the response to SHMT modulation in A549 and H1299 lung cancer cell lines [12]. SHMT1 overexpression in H1299 cells causes a strong down-regulation of SHMT2 (Supplemental Figure 4), which is less evident in A549 cells. This crosstalk between the expression of the two SHMT isoforms, particularly in H1299 cells, may have ultimately prevented a clear response with this cell line in the above experiments.

## DISCUSSION

SHMTs are key enzymes in the metabolism of several cancer cell types, including lung, colon and breast [12, 25]. They occupy a key position in the complex SGOC pathway, controlling the production of DNA bases, metabolic intermediates and antioxidant molecules that are fundamental for rapidly proliferating cells. In a recent patent application by BASF AG (WO 2013182472 A1) several pyrazolopyran compounds were disclosed as inhibitors of *Arabidopsis thaliana* SHMT activity. Moreover, a recent paper demonstrated that molecules from the pyrazolopyran series are also active against the SHMT activity of *Plasmodium falciparum*. In this work we have selected two representatives of the pyrazolopyran scaffold and tested *in vitro* their ability to block the activity of human SHMTs. Our results show that compound 2.12 behaves as an antifolate, binding to the THF site in human SHMTs, but is less active towards the human enzymes than pfSHMT. This demonstrates that, although SHMTs from different sources are structurally very similar, small differences can have a large effect on ligand and/or cofactor binding and on inhibitory potential. Our experiments also show that among human SHMT isoforms, SHMT1 is more effectively inhibited by compound 2.12 than SHMT2. Our *in vitro* data suggests that it may be possible to design pyrazolopyran-type inhibitors of human SHMT which selectively inhibit one isoform of the enzyme over the other.

The ability of 2.12 to inhibit human SHMTs was also demonstrated in living cells. Our experiments do not allow us to assess the individual contribution of the two isoforms to the overall SHMT activity in cells. However, we were able to demonstrate that treatment with 2.12 led to significant decrease of glycine levels in lung cancer cells. This result is in agreement with an inhibitory effect of 2.12 on SHMT activity. A concomitant large increase in serine concentration would also be expected, yet we observed only a small, non-significant increase of serine. We believe that this is due to serine consumption by the activity of SHMT2 in the mitochondria which is probably unaffected by 2.12, given the observed low inhibitory effect of this compound on SHMT2. Serine would therefore enter the mitochondria and be consumed in the SHMT2-catalysed conversion into glycine and ME-THF. In turn, glycine would be irreversibly oxidized to CO_2_ and ME-THF by the mitochondrial glycine cleavage system. The formate derived from ME-THF would then be exported to the cytosol where it is converted into folate-activated carbon units [27, 28]. Our previously published data demonstrate that, differently from other cancer cell types, lung cancer cells are extremely sensitive to SHMT1 levels [12]. RNAi against this protein induces apoptosis at a higher level compared to RNAi against SHMT2, which is generally considered a more suitable target for chemotherapy [25]. Considering the ability of 2.12 to preferentially inhibit human SHMT1, we treated lung and colon cancer cell types with this compound and studied the induction of cell death. We demonstrated that lung cancer cells are more sensitive to 2.12 when compared to colon cancer cells, and we suggest that this difference could be ascribed, at least in part, to the differential expression of SHMT1 in these cell lines.

A more detailed analysis demonstrated that the 2.12-induced cell death is attributable to an apoptotic signal. Rescue experiments have been previously used to confirm the ability of an inhibitor to block an enzyme in a specific metabolic process. In our experiments, supplementation of glycine, serine and 5-formyl-THF promotes a ∼30% recovery from the 2.12-induced apoptosis and suggests that 2.12 inhibits SHMT activity in living cells. Recovery to the same extent (∼about 30%) was also observed after overexpression of SHMT1 in 2.12-treated A549 lung cancer cells. This observation correlates with the results obtained with the medium supplementation experiments mentioned above. A more complex picture was observed with 2.12-treated H1299 cells in which SHMT1 was overexpressed to counteract the inhibitory effect of 2.12. In these experiments a rescue trend similar to the one observed with the A549 cells was observed, but it did not reach statistical significance. The lack of a clear rescue effect with the H1299 cell line may be due to the reduction of SHMT2 expression that has been observed in H1299, but not in the A549 SHMT1 overexpressing cells, that may alter the overall flux of metabolites in the SGOC pathway.

In conclusion, the results of this work demonstrate that 2.12 is able to induce a strong apoptotic signal in lung cancer cells. At this stage we cannot exclude that 2.12 may inhibit enzymes other than SHMT involved in the folate cycle and further studies will be carried out to evaluate other possible target(s). Moreover, the present data support the potential of a differential inhibition of the two human SHMT isozymes with compounds based on the pyrazolopyran scaffold.

## MATERIALS AND METHODS

### Compounds 2.12 and 2.2

Compounds 2.2 and 2.12 were prepared as described in WO2013/182472 A1 [22].

### Crystal structure with 2.2 and 2.12

Needle-shaped crystals were obtained by mixing equal volumes of a solution containing 50 μM human SHMT1, 40 mM glycine and 400 μM of 2.12 (or 2.2) inhibitor with a crystallization solution containing tri-potassium citrate and PEG 3350. A low-resolution (4.2 Å) complete data-set, collected at ESRF ID23.1 beamline, was processed with XDS [29] and phased by molecular replacement with MOLREP [30] using SHMT1 structure as template PDB code: 1BJ4 [15]. The space group was P212121 with unit cell constants; *a* = 140.38, *b* = 140.52, c = 268.39. The crystals were twinned with twin operator -k,-h,-l originating a pseudo tetrameric symmetry. Final model consisted of 8 mol/AU and was refined in REFMAC5 [31] to a final R_work_ and R_free_ of 32.7 and 33.7, respectively. The resolution of the electron density maps was too low to build a reliable model of the inhibitor, however a positive electron density up to 5 sigma was observed in the F_obs_−F_cal_ map.

### Protein purification

SHMT1 and SHMT2 were purified as described in [32].

### Inhibition experiments on purified recombinant SHMT isoforms

Inhibition of SHMT activity by compound 2.12 was tested using competitive binding assays in which the antifolate competes for binding with H_4_PteGlu. The first assay is based on the spectrophotometric measurement of the quinonoid intermediate that develops when both glycine and H_4_PteGlu bind to SHMT, forming an enzyme-glycine-folate ternary complex [20]. The quinonoid intermediate, which yields an intense absorption band with a maximum at around 500 nm, derives from deprotonation of glycine, but it accumulates to a measurable extent only when a folate ligand is also bound to SHMT and a ternary complex is formed [33]. Therefore, absorbance at 500 nm is proportional to the fraction of enzyme present as ternary complex. The assay was carried out at 30°C in 20 mM KPi buffer pH 7.2. Compound 2.12 was dissolved in pure DMSO. The effect of DMSO concentration on quinonoid development was analysed and found to be negligible up to 20% DMSO (v/v). In all inhibition assays, DMSO final concentration was 5% (v/v) and H_4_PteGlu was added as last component. After a rapid manual mixing, the absorbance change at 500 nm was measured. All measurements were performed in triplicate. Inhibition curves were fitted to Eq. 1, in which [*I*] is the concentration of the inhibitor, to obtain the observed inhibition constants (*Ki*).

Equation 1 $$\%\text{Activity}=100\times (1-\frac{\left[I\right]}{\left[I\right]+K_{i}})$$

Fitting of data was performed with the software PRISM (GraphPad, La Jolla, CA, USA). The second radioisotope assay was based on the capability of SHMT to catalyse the exchange of the pro-2S proton of glycine with solvent [26]. SHMT activity was determined by incubating 23 μM [2^−3^H]glycine (2 × 10^9^ dpm μmol^−1^) and 20 μM H_4_PteGlu with increasing concentrations (from 9.75 to 156 μM) of compound 2.12 in 70 μl of 20 mM potassium phosphate buffer pH 7.2. Control reactions were performed to correct for background exchange by the addition of 0.5 mM 5-CHO-H_4_PteGlu. Reactions were started by the addition of glycine and incubated at 30°C for 2 h. After incubation, reactions were stopped by the addition of 3% (w/v) trichloroacetic acid and treated as previously detailed [26] so as to remove radiolabeled glycine and measure radioactivity in the solvent. All measurements were performed in triplicate.

### Inhibition experiments on living cells

SHMT activity measurements on living cells were based on the radioisotope assay explained above. A549, H1299 and COLO320 cell lines grown in complete medium RPMI-1640 were detached using trypsin, centrifuged and suspended twice in 2 ml of PBS buffer to eliminate the growth medium; 100 μl aliquots of cell suspension were incubated with increasing concentrations of compound 2.12 (from 9 to 315 μM) at 37°C for 2 h. Glycine (23 μM) was then added to the samples, which were further incubated for 4 hours at 37°C. At this point, samples were centrifuged to remove cells, reactions were stopped by the addition of 3% (w/v) trichloroacetic acid and treated as previously detailed [26] so as to remove radiolabeled glycine and measure radioactivity in the solvent. Control reactions were performed to correct for background exchange (these samples did not contain cells) and to measure 100% of activity (these sample did not contain the inhibitor). All measurements were performed in duplicate.

### Docking of 2.12 to SHMT1

The crystal structure of SHMT1 in its biologically active tetrameric form, and in complex with PLP as internal aldimine [15], was used as a starting point to model the “closed” form of the enzyme in complex with PLP-glycine, as previously described [20]. The Dundee PRODRG2 Server was used to build the energy minimized three-dimensional structure of 2.12 [34]. Template-based molecular docking was carried out by means of Molegro Virtual Docker (MVD) software (^®^CLCbio). Flexible torsions of 2.12 were automatically detected by MVD, and manually checked for consistency. The obtained three-dimensional structure of human SHMT1 was prepared by automatically assigning bond orders and hybridization, and adding explicit hydrogens, charges and Tripos atom types. A search space of 15 Å radius, centered on the active site cavity, was used for docking. The pyrazolopyran skeleton of CAS 1508291-70-0 was taken as pharmacophoric group for template-based dockings [22]. In the latter, if an atom of the ligand matches a group definition, it is rewarded by using a weighted score that depends on its distance to the group centers. The grid-based MolDock score with a grid resolution of 0.30 Å was used as scoring function and MolDock SE was used as docking algorithm [35]. For each ligand, ten runs were defined. Similar poses (RMSD ≤ 1.0 Å) were clustered, and the best scoring one was taken as representative. Other docking parameters were fixed at their default values. After docking, energy optimization of hydrogen bonds was performed.

### Molecular dynamics

Molecular dynamics simulations were performed using version 14 of Amber with the Amber14ffsb force field. Monomeric structures of human SHMT1 (PDB 1BJ4) and SHMT2 (PDB 4PVF) were assembled into dimer structures using the fully resolved mouse dimeric structure (PDB 1EJI) as a template. Missing loops in the SHMT2 structure (distant from the active site) were built using I-TASSER [37]. Systems were solvated in a TIP3P octahedral 12Å water box and two rounds of minimization and molecular dynamics equilibration were applied prior to the production run of 100 ns. Complete simulation parameters are provided in the supplementary information.

### Cell lines

COLO320 were purchased from ATCC (Manassas, VA, USA). A549, H1299 and COLO320 cancer cells were grown in RPMI-1640 medium supplemented with 2 mM L-glutamine, 100 IU/ml penicillin/streptomycin, and 10% fetal calf serum (FCS; Sigma-Aldrich, St Louis, MO, USA). The rescue experiments were performed in A549 and H1299 cancer cells grown in Miminum Essential Medium Eagle (Sigma-Aldrich) supplemented with 1X MEM Vitamin Solution, 2 mM L-glutamine, 25 mM glucose, 10% fetal bovine serum dialyzed, 100 IU/ml penicillin/streptomycin and 0.4 mM serine, 0.4 mM glycine and 0.02 mM 5-formyl-THF (Merck Eprova AG).

### Chemicals and reagents

2-(4-chlorophenyl)ethylamine, ethyl chloroformate, diethyl ether, ethyl acetate and dichloromethane were obtained from Sigma-Aldrich.

### Sample extraction and derivatization

Serine and Glycine were analyzed according to the method of Gao et al. with slight modification [38]. Cell (A549 and H1299) pellets were suspended in 100 μl of chloroform/methanol/water (1:3:1 ratio) at 4°C and mixed vigorously to break up the pellet. After centrifugation for 3 min (13,000 g at 4°C) the supernatant was collected, dried under N_2_, resuspended in 500 μl of water and spiked with 20 μl of 0.7 mM 2-(4-chlorophenyl)ethylamine. The first step of derivatization was performed by adding 350 μl of ethanol/pyridine (6:1) and 50 μl of ethyl chloroformate (ECF). The reaction mixture was then vortexed for 30 sec and ultrasonicated for 60 sec to increase the speed of reaction at room temperature. The derivatization products were extracted with 700 μl of *n*-hexane for 60 sec and centrifuged for 5 min at 3000 rpm. The organic layer was removed and the aqueous phase was adjusted to pH ≥12 with 100 μl 7M NaOH. The second derivatization step was performed by adding further 50 μl of ECF and 700 μl of n-hexane. The reaction mixture was again vortexed for 30 sec, ultrasonicated for 60 sec, and centrifuged at 3000 rpm for 10 min. The organic layers were combined, dried under *N*_2_, resuspended in dichloromethane and analyzed by GC/MS.

### Gascromatography-mass spectrometry

GC-M analyses were performed with an Agilent 6850A gas chromatograph coupled to a 5973N quadrupole mass selective detector (Agilent Technologies, Palo Alto, CA, USA). Chromatographic separations were carried out with an Agilent HP5ms fused-silica capillary column (30 m × 0.25 mm i.d.) coated with 5%- phenyl-95%-dimethylpolysiloxane (film thickness 0.25 μm) as stationary phase. Injection mode: splitless at a temperature of 280°C. Column temperature program: 80°C (2 min) ramped to 140°C at a rate of 10°C/min, to 240°C at a rate of 4°C/min, to 280°C at a rate of 10°C/min and held for 5 min. The carrier gas was helium at a constant flow of 1.0 ml/min. The spectra were obtained in the electron impact mode at 70 eV ionization energy; ion source 280°C; ion source vacuum 10^−5^ Torr. MS analysis was performed simultaneously in TIC (mass range scan from m/z 50 to 600 at a rate of 0.42 scans s^−1^) and SIM mode. GC-SIM-MS analysis was performed selecting the following ions: m/z 102 for glycine, m/z 114 for serine and m/z 227 for 3, 4-dimethoxybenzoic acid (internal standard).

### RNA extraction and real-time qRT–PCR analyses

Total RNA was extracted using TRIzol reagent (Invitrogen) following the manufacturers instruction and 1 μg was used for the reverse transcription reaction by using a SuperScript First-Strand Synthesis System Kit (Invitrogen). One microliter of the 1:50 dilution of the complementary DNA was used for qRT–PCR analysis performed in triplicate for each sample using KAPA Sybr Fast universal qPCR Kit following the manufacturer instructions. Reactions were performed by Stratagene MX3000P (Stratagene, La Jolla, CA, USA). Primers used have been previously reported [12].

### Western blot analysis

Cell lysates were prepared in 8 M urea (Sigma-Aldrich) and protein concentration was determined by Biorad Protein Assay (Bio-Rad, Munchen, Germany). Lysates were subjected to SDS–polyacrylamide gel electrophoresis and were transferred onto a nitrocellulose membrane then saturated with 5% non fat dry milk in Tris-buffered saline with 0.1% Tween-20 for 1 h at room temperature. Membranes were incubated with primary antibody overnight at 4°C and subsequently with horseradish peroxidase-conjugated secondary antibody for 1 h at room temperature. Membranes were washed with Tris-buffered saline with 0.1% Tween-20 and ECL reagent (Millipore) was used for detection by chemiluminescence system Chemidoc MP Imaging System (Bio-Rad). Antibody anti-SHMT1 were from Cell Signaling Technology (Danvers, MA, USA), the secondary antibody anti-rabbit were from Abbiotec (San Diego, CA, USA), antibody anti-SHMT2, -β-actin and the secondary antibodies anti-goat and -mouse were from Santa Cruz Biotechnology (Santa Cruz, CA, USA).

### Trypan blue exclusion assay

The cells were harvested and washed in cold PBS two times, following the addition of a 0.4% (w/v) trypan blue solution (Sigma-Aldrich), cells were counted using a Burker chamber (Hirschmann, Germany) under an Axioskop 2 plus microscope (Carl Zeiss Microscopy, Switzerland). Cells staining with trypan blue dye were counted as nonviable.

### XTT assay

In order to determine the percentage of viable cells this assay employs 2, 3-Bis-(2-methoxy-4-nitro-5-sulfophenyl)-2H-tetrazolium-5-carboxanilide salt (XTT). Only in living cells are mitochondria capable of reducing XTT to form an orange colored dye. Therefore, the concentration of the dye is proportional to the number of metabolically active cells. Approximately 1200 cells/well were plated in 96-well dishes in complete medium RPMI-1640. After 24 h, 48 h and 72 h cells were treated for 72 h, 48 h and 24 h by adding complete medium and 2.12 compound at different concentrations. Each test includes a blank containing complete medium without cells and a negative control made up of 2.12 untreated cells. XTT mix reaction (AppliChem GmbH, Darmstadt, Germany) was prepared and then added to cell cultures according to the manufacturer's recommendations. After 3 h in CO_2_ incubator at 37°C, the absorbance of samples was measured by a spectrophotometer Appliskan Multimode Microplate Reader (Thermo Scientific, Waltham, MA USA) at a wavelength of 470 nm. In order to measure non-specific reading the absorbance of samples was measured also at a wavelength of 690 nm and then subtracted from the 470 nm measurement to measure reference absorbance. The percentage of metabolically active cells was calculated according to the following formula ((Abs sample – Abs Blank)/(Abs Neg Control – Abs Blank)) × 100.

### Apoptosis assay

For PI staining, cells were detached with trypsin, washed with cold PBS plus 5% FCS and then fixed in 70% ethanol for 24 h. After washing with PBS, cells were incubated with 1 μg/ml PI for 3 h at 25°C before cytometric analysis by BD Accuri C6 Flow Cytometer (Becton Dickinson, Franklin Lakes, NJ, USA). Results were analyzed with BD Accuri C6 software and were presented as a percentage of specific apoptosis determined using the following formula: ((% apoptotic cells in experimental sample–% apoptotic cells in control sample)/(100–% apoptotic cells in control sample) × 100). For AnnexinV staining: cells were detached with trypsin, washed with PBS–5% FCS and then placed in binding buffer (Pharmingen, San Diego, CA, USA) to which 7-amino-actinomycin D (7-AAD) and AnnexinV-APC (Pharmingen) were added prior to cytometric analysis. Cells were considered apoptotic when Annexin-V-APC positive and 7-AAD negative.

### Cell transfection

Transient transfection experiments were carried out by seeding cells (1 × 10^5^ cells/plate) in 6-well dishes in complete medium RPMI-1640. Transfection was performed adding equal amount of pCDNA and pCDNA_SHMT1 (pSHMT1) plasmids 24 h after plating using Lipofectamine 2000 reagent in Optimem medium (Invitrogen). Optimem medium with plasmids was replaced with complete medium RPMI-1640 after 6 h and the evaluation of protein expression levels was performed by harvesting cells at 48 h after transfection.

### Statistical analysis

Statistical differences were determined either by Student's *t*-test for paired samples or by one-way analysis of variance followed by Student's *t*-test with the Bonferroni correction. *P* ≤ 0.05 was considered significant.

## SUPPLEMENTARY MATERIALS FIGURES


