# Supplementary material for: A pyrazolopyran derivative preferentially inhibits the activity of human cytosolic serine hydroxymethyltransferase and induces cell death in lung cancer cells
Source: Oncotarget. 2015 Dec 22;7(4):4570–83. doi: 10.18632/oncotarget.6726 (PMC4826227; doi:10.18632/oncotarget.6726)
Supplement: Supplementary file 1 [file oncotarget-07-4570-s001.pdf]

A pyrazolopyran derivative preferentially inhibits the activity of human cytosolic serine hydroxymethyltransferase and induces cell death in lung cancer cells

Supplementary Materials

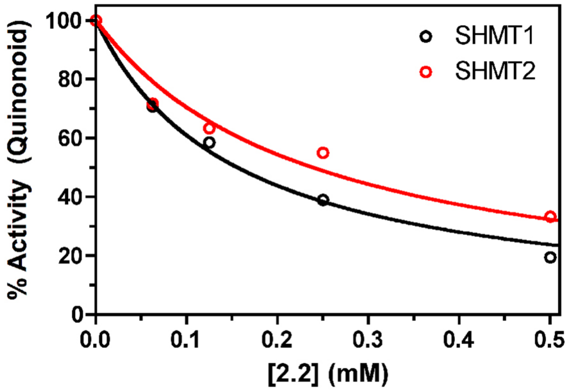

Supplementary Figure S1: Inhibition of SHMT isoforms by compound 2.2. The activity of SHMT1 (black circles) and SHMT2 (red circles) was measured in the presence of increasing concentrations of compound 2.2. Continuous lines through the experimental points are those obtained from the least square fitting of data to equation 1. The assay was based on the spectrophotometric measurement of the quinonoid intermediate, as in Figure 2A. Estimated inhibition constants were 154.5 ± 14.4 µM for SHMT1 and 262.8 ± 48.0 µM for SHMT2.

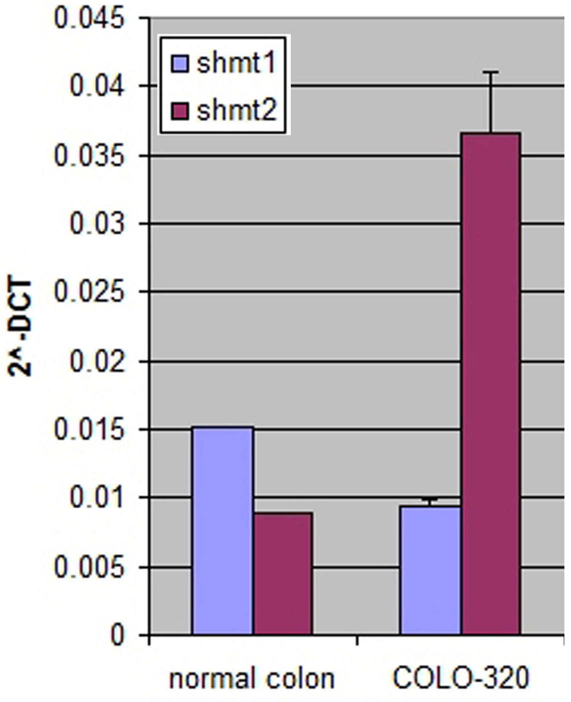

Supplementary Figure S2: SHMT1 and SHMT2 mRNA expression in colon cancer cell line. Expression levels of SHMT1 and SHMT2 mRNA in COLO320 cell line compared with normal colon sample as measured by quantitative real-time PCR (qRT-PCR). All data represent mean ± standard deviation of at least 3 independent experiments.

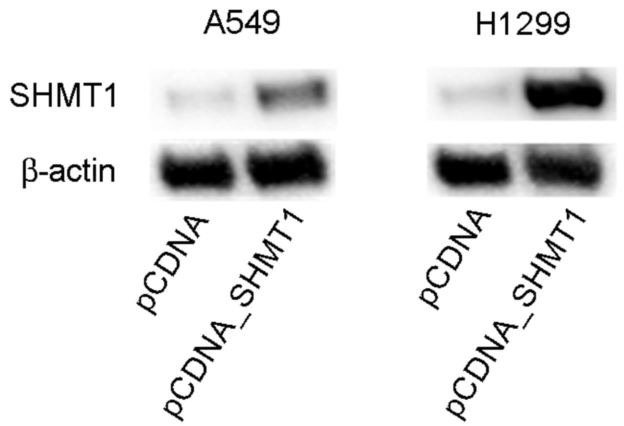

Supplementary Figure S3: Overexpression of SHMT1. Western blot analysis for SHMT1 protein expression performed on cellular extract from A549 and H1299 cells transfected with the indicated plasmid, β-Actin has been used as loading control.

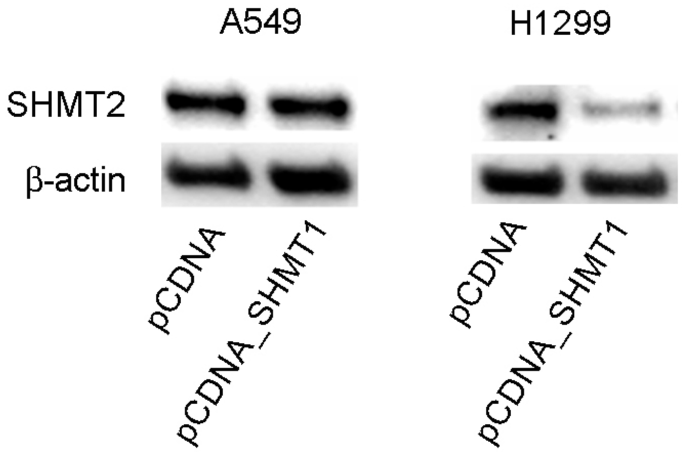

Supplementary Figure S4: Effect of SHMT1-overexpression on SHMT2 levels. Western blot analysis for SHMT2 protein expression performed on cellular extract from A549 and H1299 cells transfected with the indicated plasmid, β-Actin has been used as loading control.

Parameters for Amber MD simulations

```
tleap input (after startup with –f leaprc.ff14SB):
prot=loadpdb prot.pdb
loadamberparams “frcmod.ionsjc_tip3p”
solvateoct prot TIP3PBOX 12
addions prot Na+ 0
addions prot Cl− 0
saveamberparm prot prot.prmtop prot.inpcrd
quit

initial minimization solvent + ions
 &cntrl
  imin   = 1,
  maxcyc = 1000,
  ncyc   = 500,
  ntb    = 1,
  ntr    = 1,
  cut    = 10.0
 /

Hold the protein fixed
500.0
FIND
* * S *
* * B *
* * 3 *
* * E *
* * M *
SEARCH
RES 1 924
END
END

minimization whole system
 &cntrl
  imin   = 1,
  maxcyc = 5000,
  ncyc   = 2000,
  ntb    = 1,
  ntr    = 0,
  cut    = 10.0
 /

warm restrained system
&cntrl
  imin   = 0,
  irest  = 0,
  ntx    = 1,
  ntb    = 1,
  cut    = 10.0,
  ntr    = 1,
  ntc    = 2,
  ntf    = 2,
  tempi  = 0.0,
  temp0  = 300,
  ntt    = 3,
  gamma_ln = 1.0,
  nstlim = 50000, dt = 0.002, ntxo = 2,
  ntpr = 1000, ntwx = 1000, ntwr = 10000,
  ioutfm = 1
 /

Keep prot fixed with weak restraints
10.0
FIND
* * S *
* * B *
* * 3 *
* * E *
* * M *
SEARCH
RES 1 924
END
END
```

100ps MD **equilibration**

```
 &cntrl
  imin = 0, irest = 1, ntx = 7,
  ntb = 2, pres0 = 1.0, ntp = 1,
  taup = 2.0,
  cut = 10.0, ntr = 0,
  ntc = 2, ntf = 2,
  tempi = 300, temp0 = 300,
  ntt = 3, gamma_ln = 1.0,
  nstlim = 50000, dt = 0.002, ntxo = 2,
  ntpr = 5000, ntwx = 5000, ntwr = 500000,
  ioutfm = 1
 /

100ns production MD
 &cntrl
  imin = 0, irest = 1, ntx = 7,
  ntb = 2, pres0 = 1.0, ntp = 1,
  taup = 2.0,
  cut = 10.0, ntr = 0,
  ntc = 2, ntf = 2,
  tempi = 300, temp0 = 300,
  ntt = 3, gamma_ln = 1.0,
  nstlim = 50000000, dt = 0.002, ntxo = 2,
  ntpr = 5000, ntwx = 500, ntwr = 500000,
  ioutfm = 1, ig = −1
  /
```
